# Comparing Robotic and Real Cat Companionship in Mitigating Loneliness Among Elderly Populations

**DOI:** 10.1093/geroni/igaf122.2245

**Published:** 2025-12-31

**Authors:** Bingwen Wang, Kaiting Ma

**Affiliations:** Duke University, Durham, North Carolina, United States; The Education University of Hong Kong, Hong Kong, China (People’s Republic)

## Abstract

**Aims:**

This study evaluates the effectiveness of a newly developed robotic cat compared to live cat therapy in reducing loneliness among elderly individuals. It aims to determine whether robotic feline companionship can provide similar emotional and mental health benefits to real cat interactions, offering a scalable solution for socially isolated older adults.

**Method:**

A randomized controlled trial was conducted with 180 elderly participants experiencing chronic loneliness. Participants were assigned to one of three groups: interaction with a robotic cat, interaction with a real therapy cat, or a control group receiving standard care. The study lasted six months, with outcomes measured using the UCLA Loneliness Scale, Geriatric Depression Scale, and physiological stress indicators (cortisol levels and heart rate variability). Semi-structured interviews explored participants’ subjective experiences and attachment to the cats.

**Results:**

Both intervention groups showed significant improvements in loneliness and mental health compared to the control group. The robotic cat group experienced a 38% reduction in loneliness scores and a 28% improvement in mental health metrics, while the real cat group reported a 45% reduction in loneliness and a 35% improvement in mental health. Qualitative feedback indicated that robotic cat users valued its constant availability and ease of care, while real cat users highlighted emotional warmth and authenticity.

**Conclusion:**

The robotic cat presents a promising alternative to live cat therapy, significantly reducing loneliness and enhancing well-being. Its practicality and accessibility make it a viable, scalable intervention, though further research is needed to optimize design and assess long-term effects.

